# Newly qualified registered nurses’ and midwives’ experiences from rural health district placement in Namibia

**DOI:** 10.1186/s12912-023-01272-2

**Published:** 2023-04-07

**Authors:** Martha N Katuta, Vistolina Nuuyoma

**Affiliations:** School of Nursing and Public Health, Faculty of Health Sciences and Veterinary Medicine, University of Namibia, Rundu Campus, Rundu, Namibia

**Keywords:** Newly qualified registered nurse/midwife, Newly qualified nurse/midwife, Nurse transition, Qualitative study, Role transition, Rural health, Rural community, Social adaptation, Transition

## Abstract

**Background:**

The transition period for newly qualified registered nurses/midwives (NQRN/Ms) is a fundamental phase in their career. Yet, transition experiences have been studied mostly within urban and/or specialised healthcare settings in high-resource countries. This study aimed to explore and describe the experiences of NQRN/Ms in a rural health district in Namibia.

**Methods:**

A qualitative, descriptive, explorative, and contextual design was followed. The sample consisted of eight participants who were purposively selected. Data were collected via in-depth individual interviews and analysed following a reflexive thematic analysis. The researchers were guided by Lincoln and Guba’s strategies for establishing trustworthiness.

**Findings:**

Themes conceptualised from the analysis include encounters with rural community members; encounters with colleagues; staffing, management, and supervision; shortage of resources; poor infrastructure; unreliable communication networks; and the lack of social life.

**Conclusion:**

The NQRN/Ms had mixed experiences related to a variety of aspects such as social life, resources, colleagues and community members. These findings can be used to improve undergraduate nursing curricula, as well as to create graduate job preparation workshops and support networks.

**Supplementary Information:**

The online version contains supplementary material available at 10.1186/s12912-023-01272-2.

## Introduction

Newly qualified registered nurses, also known as newly graduated registered nurses, have work experience of less than one year after registration [1]. In contexts where midwifery and nursing training are offered as a comprehensive programme, graduates are referred to as newly qualified registered nurses/midwives (NQRN/Ms). During nursing training, students are expected to work under the direct supervision of qualified registered nurses. These nurses guide them and facilitate their learning regarding the necessary skills, behaviour, and knowledge they need to become critical thinkers who are competent at delivering safe and high-quality patient care [2]. In this way, NQRN/Ms are in a transition period during which they develop from supervised nursing students to independent registered nurses and midwives.

This transition period has been identified as a fundamental phase in the careers of NQRN/Ms, as it either positively contributes to their engagement in their new roles or inspires the intention to leave the profession [3]. NQRN/Ms often doubt their own competence upon graduating as they realise that they need to acquire a vast range of skills to fill different roles successfully and to guarantee a high standard of nursing care [4]. NQRN/Ms should thus be adequately supported during this period. A rapid evidence assessment by Wray et al. [5] revealed that both formal and informal approaches are used to enhance the transition of newly qualified nurses. Formal approaches used include educational preparation, mentorships, internships, preceptorships, and simulations. The presence of a supportive organisational culture together with the provision of learning opportunities and constructive feedback are considered informal approaches to enhance role transition.

According to Jarden et al. [6], NQRN/Ms experience higher job satisfaction after 12 months, which is positive when it comes to their work well-being. However, they also report high levels of emotional exhaustion and stress as a result of a formidable workload and the incivility of their co-workers and supervisors. These stress levels are related to the transitory levels of high task mastery, role clarity and social acceptance. During their first year of work, NQRN/Ms fully develop their professional and personal identity through the support – or lack thereof – of their colleagues and preceptors [7]. The experiences of NQRN/Ms during this transition period involve challenges such as negotiating the differences between theory and practical applications [8]; occupational stress [9]; exposure to negative behaviours [10]; difficult working and environmental conditions; and fear and uncertainty regarding living up to one’s own and others’ expectations [7].

Globally, most studies conducted on the experiences of NQRN/Ms during the transition period have focused on private healthcare facilities in urban settings, mainly in developed and industrialised countries [4, 8], while others have been review studies [3, 6, 7, 9, 11, 12]. However, the experiences of NQRN/Ms in developed and industrial countries, urban settings and private healthcare facilities may vary from those in the rural areas of a developing country owing to differences in available resources, infrastructure and the type of healthcare services offered. Some studies on the experiences of NQRN/Ms in rural areas have focused specifically on caring for deteriorating patients [12], while others have focused on specific nursing disciplines; for example, Taylor and Foster [7] examined the experiences of newly graduated nurses working in a paediatric setting, while Elias and Day [13] investigated the experiences of newly qualified nurses in critical care units. These experiences may not be generalisable to NQRN/Ms in generic rural health district hospitals.

In Namibia, nursing training is offered as a comprehensive programme which consists of midwifery and nursing courses. Thereafter, NQRN/Ms are placed in rural and urban settings to promote universal health coverage. This coverage enables individuals and communities to receive essential health services without enduring financial hardship [14]. Despite that, up to now it has not explicitly been known how NQRN/Ms experience placement in a rural health district in Namibia.

Moreover, in Namibia and many parts of the world, nursing schools are located mainly in urban settings, with limited numbers of students being exposed to rural health facilities. Nursing students may subsequently be placed in rural health clinics and health centres and not in district hospitals where in-patient care services are offered. In some settings, nursing students’ rural health placements focus largely on home visits, school visits and talking to community members [15]. Therefore, the focus of this article is on exploring and describing how NQRN/Ms in a rural health district experience placement during their transition period. This is important for identifying gaps in undergraduate training curricula, establishing evidence-based transition programmes in workplaces, and improving graduate retention strategies. This may indirectly assist in achieving the global competency needed for universal health coverage [14].

## Methods

### Study design

The study utilised a qualitative, descriptive, explorative, and contextual design. This was necessitated by the need for an in-depth description and understanding of NQRN/Ms’ experiences and events in their real-life situations [16]. This design follows the assumptions of a constructivist philosophical research paradigm. The study used the Consolidated Criteria for Reporting Qualitative Research (COREQ) as a reporting guideline [17].

### Study setting

The setting for the study was a rural district located 293 km northeast of Windhoek, the capital city of Namibia. This district has five primary healthcare clinics and a district hospital with 86 beds and 59 nurses and midwives.

### Study population and sample

The targeted population for this study was NQRN/Ms, which within the context of this study refers to registered nurses/midwives who have recently completed undergraduate training and have been employed for less than two years. At the time of data collection, the rural district in question had a total of 13 NQRN/Ms. Every year, the district receives an average of 10 NQN/Ms. There are usually more vacancies in this district than in others owing to the introduction of a new programme, the extension of departments, transfers to other districts and resignations. Participants were purposively selected using the following inclusion criteria: the NQRN/Ms were placed in this specific rural health district on completion of their undergraduate training; they were willing to participate in the study; and they gave written informed consent. One NQRN/M was excluded as this nurse had initially been placed in another district.

The first author contacted the district hospital nurse manager to obtain the contact details of all the NQRN/Ms and the units in which they were placed. All the NQRN/Ms were contacted either telephonically or in person to request their participation in the study. At that time, the aim of the study was explained to them and they were given a participant information sheet. Of all the NQRN/Ms who were approached to participate in the study, none refused or dropped out. Data saturation was reached with eight participants; meaning that no new analytical information could be gleaned from further interviews and the researchers judged that sufficient information on the phenomenon had been gathered [18].

### Data collection procedures

Data were collected during November and December 2021. In-depth individual interviews were carried out with the eight participants, during which an interview guide was used. This guide was initially piloted with two participants prior to the main data collection process. A pilot study was necessary to ensure high research quality in terms of understanding NQRN/Ms’ experiences [19]. The central question posed to all participants was: *“What is your experience of placement in a rural health district as a newly qualified registered nurse/midwife?”* This was followed by probes and prompts to facilitate further exploration and understanding of the participants’ responses. These helped the interview process to remain unstructured and flexible, as well as to create a dialogue [18]. With the participants’ written consent, all the interviews were audio recorded using a smartphone. The interviews were conducted by the first author and no other people were present during the interview. The interviews took place face to face in various rooms at the district hospital in the rural health district. In all cases, the researcher and the participants reached consensus on the date, time and venue for each interview. English was used to conduct the interviews as it is an official language of Namibia, and all the participants were comfortable conversing in it. The interview protocol is attached as supplementary material two.

Field notes, which consisted of notes on body language, nonverbal communication, the researcher’s reflection, and the bracketing of the researcher’s experiences, were made during and after each interview. The interviews ended when no new information emerged and the interviewer judged that all probes noted down had been answered, i.e. data saturation had been reached. The duration of the interviews ranged from 40 to 55 min. The interview recordings were transcribed verbatim within 24 h of each interview and were shared with the respective participants for member checking.

### Data analysis

The data were analysed manually by the two authors, following a reflexive thematic analysis approach [20]. This six-phase process consisted of data familiarisation and writing familiarisation notes; systematic data coding; generating initial themes from coded and collated data; developing and reviewing themes; refining, defining, and naming themes; and writing the report. The field notes were read together with the transcribed data during the data analysis process. The first author designed a coding tree that displayed the themes and explained how the sub-themes emerged as well as the codes that formed them. This was necessary for understanding how the themes were generated and provided evidence that the themes were derived from the data, not identified in advance. Finally, the two authors agreed on the themes and sub-themes for reporting. An inquiry audit was undertaken by an external reviewer who checked the coding tree, field notes and transcribed data to ensure that the themes had been extracted from the collected data. The external reviewer in this case was a nurse educator with a Masters in Nursing Science and extensive experience in qualitative research.

### Study quality criteria

The trustworthiness of the study was ensured by following the four principles of Lincoln and Guba [21]. In addition, reflexivity was ensured. Credibility was ensured by piloting the interview guide, collecting data until saturation was achieved, prolonged engagement with the participants, audio recording all the interviews and member checking. Transferability was ensured by including rich descriptions of the NQRN/Ms’ experiences and their contexts, so that they are meaningful to an outsider [22]. Peer debriefing with other researchers, prolonged engagement and member checking ensured dependability, while an inquiry audit through the use of an external reviewer was used to ensure conformability. Lastly, to ensure reflexivity, the researchers remained conscious of the part they were playing in the study and continuously reflected on their own behaviours and how they might affect the study [23]. This was done through the use of research diaries, which were included in the field notes.

### Ethical considerations

Research was performed in accordance with the Declaration of Helsinki. In addition, all participants gave their informed consent by signing a form prior to participation in the study. Participation was voluntary and the interviewees were told that there would be no coercion if they wanted to opt out of the study. The interviewees did not have to answer any questions they were not comfortable with and could also opt out of the study at any point with no penalties. No form of incentive was used during the recruitment process and inclusion criteria were met to ensure impartiality Anonymity was assured by allocating numbers to the participants instead of using their names. The audio recordings were stored on password-protected devices and only the researchers had access to them, which ensured confidentiality. The study was approved by the university’s departmental research ethics committee on 1 September 2021. In addition, ethical clearance was granted by the research unit in the Ministry of Health and Social Services on 28 October 2021.

## Findings

The demographic characteristics of the eight participants are shown in Table 1.

**Table 1:** Demographic characteristics of the participants

Participant number	Sex	Age in years	Marital status	Nursing training institutions	Prior experience in healthcare settings	Work experience as an NQRN/M
1	Male	28	Single	Public university, main campus	No	18 months
2	Female	27	Single	Public university, main campus	No	7 months
3	Male	26	Single	Public university, main campus	No	12 months
4	Female	24	Single	Public university, north-western campus	No	9 months
5	Female	25	Single	Public university, main campus	No	16 months
6	Male	23	Single	Public university, north-eastern campus	No	12 months
7	Female	25	Single	Public college, southern campus	No	21 months
8	Female	34	Married	Public university, main campus	Yes	6 months

The reflexive thematic analysis resulted in five main themes and 14 sub-themes being extracted, which are presented in Table 2. A detailed record of data analysis from quotations to themes is attached as supplementary material one.

**Table 2:** Themes and sub-themes

Main themes	Sub-themes
1. Encounters with rural community members	1. Culture shock 2. Language barrier 3. Labelled as outsiders
2. Encounters with colleagues	1. Adequate support and teamwork 2. Unrealistic expectations
3. Staffing, management and supervision	1. Insufficient nursing staff and lack of ancillary staff 2. NQRN/Ms challenged with leadership skills 3. Inadequate supervision
4. Shortage of resources and infrastructure, and unreliable communication networks	1. Shortage of equipment and clinical supplies 2. Power outages 3. Unsafe and bad roads
5. Social life	1. Feelings of loneliness and isolation from friends and family members 2. Financial burden 3. No opportunities for personal growth

### Theme 1: encounters with rural community members

This theme describes the participants’ experiences of their interactions with the residents of the rural district in which they were placed. This includes the community members’ attitudes and behaviours towards the NQRN/Ms while they were working in the district. In addition, it includes some observations made by the NQRN/Ms regarding the practices of community members during their visits to the healthcare facility.

#### Culture shock

As NQRN/Ms are sent to work in any healthcare facility where there is a vacant position, they may find themselves working with community members who are from different cultural backgrounds, which can cause culture shock. This can lead to uncertainty among NQRN/Ms as they struggle to understand the practices and behaviours of certain community members:

*I was trained in another region and was never exposed to these people, so when I came to this area it was people from a different tribe that I have to deal with. I don’t know their cultural background, I don’t know what they like and what they dislike, so it was a big shock for me.* [Participant 2]

#### Language barrier

Language barriers were mentioned by the participants, who noted that community members spoke a different language to them. Furthermore, even though some community members were able to express themselves in English, they refused to do so as they expected the nurses to be able to speak the local language:

*Yeah, the challenge here, these people, I just want to be honest, you’ll find someone … the person is young, you can see this person can communicate in English but when you’re talking to her … she’s responding in her language. Like I ask her in English, “How can I help you today?” She responded, “I know you’re here for work, but you should talk our language.” Imagine this is coming from a person who can speak English, but she does not want to.* [Participant 3]

#### Labelled as outsiders

The participants mentioned that some actions and behaviours of community members indicated that they were being labelled as outsiders, which made them feel as if they did not belong:

*The negative experiences would come from the community, not my colleagues ... Community members make me feel like I don’t belong here, because most of us that are placed here come from other districts; we are not originally from here. So even before you open your mouth, or start to help the patient, they already have a negative mindset against you and so that’s a very big disadvantage as a new person that comes here.* [Participant 5]

### Theme 2: encounters with colleagues

This theme describes the experiences of the participants in terms of their encounters with their colleagues. This includes all the health professionals and ancillary workers in the healthcare setting.

#### Adequate support and teamwork

The participants described feeling welcomed and supported by their colleagues who worked in the same department as them. This included receiving them warmly on their first day, being taught what they needed to know and being encouraged to ask questions when necessary:

*I was well received; my colleagues gave me a detailed geographical orientation around the facility and also took me through some work procedures; I felt welcomed, I felt very welcomed.* [Participant 7]

*There is good teamwork here; everyone is approachable and so friendly, even if you go to other departments in the hospital, people are friendly and work well together.* [Participant 4]

#### Unrealistic expectations

Some participants stated that certain colleagues had unrealistic expectations of the NQN/Ms, for example when they were not able to perform some procedures, and also delegated tasks to them that were too advanced for them:

*Enrolled nurses had very high expectations from me which made me a bit uncomfortable, I must say.* [Participant 8]

### Theme 3: staffing, management, and supervision

This theme describes the participants’ experiences with staffing, management, and supervision in the rural health district.

#### Insufficient nursing staff and lack of ancillary staff

The participants revealed that in most cases they were expected to work alone or with a few nursing staff even though the nursing units were fully occupied by patients. This made them feel overworked and they did not have adequate breaks in which to rest during their shifts:

*I felt so disappointed, like how to do you let a nurse work alone in a whole facility which you know that all nearby villages will be coming to seek services there and I was alone doing everything, immunisations, initiations, family planning, treating, screening, you know all these things. It was really, I had a very terrible experience. I went to report, and they told me there’s nothing they can do about it, nurses are not enough.* [Participant 7]

The participants further explained that owing to a lack of ancillary staff they had to undertake certain duties that are not supposed to be done by a nurse:

*Basically, here I am working as a porter. Had to transport patients like from maternity department to outpatient department. Sometimes there’s a body that you have to transport to the mortuary in the absence of the mortuary assistant and so on and we also do cleaning, as in some shifts there are no cleaners allocated, so it’s just too much.* [Participant 8]

#### NQRN/Ms challenged with leadership skills

The NQRN/Ms described feeling challenged as they did not have the necessary skills to lead a team or perform daily leadership-related activities:

*Although other categories like enrolled nurses are lower ranked than registered nurses, they worked more years than me and have accumulated a lot of experience. It is a problem managing them because I am still getting used to do off-duties schedule, delegation, and many others.* [Participant 7]

#### Inadequate supervision

The participants felt that they were left to work alone or with other junior nursing staff members without the supervision of a more experienced colleague. This made them feel unsafe and worried about making mistakes or causing problems for the patients, the hospital and themselves:

*When I started working here, we were in the ward, all new … we were two registered nurses but all working our probation period and no one to supervise.* [Participant 3]

### Theme 4: shortage of resources and infrastructure, and unreliable communication networks

This theme describes the experiences of the participants regarding resources, infrastructure and communication networks in the rural health district and surrounding areas.

#### Shortage of equipment and clinical supplies

The participants described having negative experiences as a result of a shortage of the equipment and clinical supplies needed to render healthcare services in the rural health district:

*We experience shortages of items like blood pressure monitoring machines, weighing scales, stethoscopes, and others. Secondly resources such as medicine, there’s literally every second day there’s something that is not in stock, it’s a real struggle.* [Participant 7]

#### Power outages

Participants revealed that the rural health district experienced a lot of power outages, which affected their work at the hospital and clinics, as well as their personal lives:

*Our electricity goes off a lot and is not good at all. When there is no electricity, the telephone network also doesn’t work that means you cannot reach to the doctor on call if there is an emergency at the hospital. You also can’t reach to the driver who is on call if you want assistance to get for you the doctor on call to come attend to emergency case.* [Participant 3]

#### Unsafe and bad roads

Participants described their experiences with the roads in the local and neighbouring districts, which were described as unsafe because they are not regularly serviced and are unsuitable for all types of vehicle:

*The roads are not well constructed and perhaps not serviced from time to time? They get worse during rainy seasons when some of the areas are not accessible.* [Participant 6]

### Theme 5: social life

This theme describes the participants’ experiences regarding their social lives, including how working in the rural health district affected them.

#### Feelings of loneliness and isolation from friends and family members

Participants explained that the rural health district has no places for leisure activities and entertainment for young people. This led to feelings of loneliness, considering that the NQRN/Ms lived far from their family members and friends:

*I cannot go visit my family members, I am staying very far, I don’t have friends, so psychologically also I am affected. If I have problems, I cannot have anyone to talk to or maybe yeah, or maybe some therapy like maybe I need to go a movie theatre just to relax my mind, it’s not available.* [Participant 6]

#### Financial burden

Participants indicated that being far away from their homes and families causes a financial burden as it requires them to spend a lot on transportation in order to visit their homes:

*I feel I have extra financial burdens because if I have to travel, I have to spend a lot on transport.* [Participant 8]

#### No opportunities for personal growth

Owing to the remoteness of the rural health district, as well as other challenges such as unreliable telecommunication networks, unsafe roads, a lack of higher education institutions and limited social interactions with other young people, the NQRN/Ms indicated that there were no opportunities for personal growth:

*News here reaches us even after three days, sometimes we don’t even view other people’s WhatsApp statuses because network trips a lot.* [Participant 3]

## Discussion

### Encounters with rural community members

The current study was conducted to explore and describe the experiences of NQRN/Ms during their placement in a rural health district. The first theme extracted was ‘Encounters with rural community members’, which emphasises the concerns around culture shock, language barriers and being labelled ‘outsiders’. The nursing staff are expected to work with community members in order to carry out successful public health interventions [24], which necessitates good relationships between the community members and nurses. Unfortunately, these relationships are challenged by issues such as culture shock, language barriers and being labelled as ’outsiders’.

Upon completion of their training, nursing graduates are placed in health districts where there are nursing shortages. This practice leads to some NQRN/Ms being placed in unfamiliar contexts, which expose them to new cultures. However, these graduates may lack awareness of the cultural practices, norms, values and beliefs of inhabitants of the districts where they are placed. As Namibia is a multicultural society with more than 20 cultural groups and languages, culture shock and language barriers should be expected. Although nurses are prepared for and introduced to transcultural care and cultural competence during their training, feelings related to culture shock are still experienced when they are exposed to unfamiliar contexts. Similar findings were reported by Adamson [25], who revealed that participants found cultural differences noticeable and surprising. Language barriers between nurses and patients may also lead to problems such as the misinterpretation of information during counselling and health education sessions, delayed treatment and medication errors [26].

Labelling, stereotypes and separation characterises stigma in healthcare settings. Although stigma may be experienced in all spheres of life, in healthcare settings people living with a specific disease or health condition can become victims of stigma [27]. In the current study, however, the NQRN/Ms alluded to cases where they felt labelled by community members because they were seen as outsiders who were not part of the community. This may have negatively affected the performance of their duties and could have resulted in them resigning from their posts and profession. The findings of the current study on the labelling of NQRN/Ms are comparable to those of Wei, Woo and Andrew [28], who revealed that new nurses experience being labelled as new, which leads them to be stigmatised by both patients and relatives.

### Encounters with colleagues

The NQRN/Ms in the current study described the presence of adequate support and good teamwork, however they were subjected to the unrealistic expectations of their colleagues. In the literature, support for NQRN/Ms is described as a formal and informal functional social structure, as well as reassurance that is usually offered by hiring organisations to facilitate the transition of nurses into new roles through interconnectedness [29]. According to Schmitt and Schiffman [29], preceptorship or the allocation of mentors is perceived to be the most important formal support and is necessary for the successful placement of newly qualified nurses. However, the current study revealed that support in the current study entailed receiving NQRN/Ms warmly into the department on their first day when reporting for duty, being taught and feeling free to ask questions when there was a need. No reference was made to the allocation of mentors or preceptors to guide the NQRN/Ms in the rural health district.

The current findings on adequate support and teamwork are consistent with Ho, Stenhouse and Snowden [30], who reported positive experiences, good teamwork and informal support being given to NQRN/Ms by their colleagues. Opposite findings were reported by Graf et al. [31], who indicated that NQRN/Ms were not being helped even when they asked for assistance from senior nurses. Moreover, the same authors reported that NQRN/Ms were ridiculed and belittled, and received too much critical feedback, which are characteristics of an unsupportive work culture.

The NQRN/Ms also experienced unrealistic expectations from the other nurses and doctors, which is in line with Labraque, McEnroe-Pettite and Leocadio’s [32] findings. These authors reported that the NQRN/Ms felt the expectations of them were too high, specifically regarding their ability to handle patients and perform certain procedures. Similarly, Wei et al. [28] reported that new nurses were expected to work efficiently, perform clinical skills independently and have skills that are specifically related to a specialised field of nursing. Considering that specialised skills are too advanced and not included in the basic curricula of nursing training, this was perceived as unrealistic.

### Staffing, management and supervision

The NQRN/Ms in the current study revealed their experience in the rural health district as challenging owing to a lack of nursing and ancillary staff. This led to the NQRN/Ms sometimes working alone or with too few nursing staff, which was not sufficient to provide care for the number of patients in a unit. This, combined with too few rest periods, led to exhaustion. This finding is not surprising as Namibia has just 19 nurses per 10,000 people [33], which is far below the global average of 36 nurses per 10,000 people. The lack of nursing staff has been previously found to create challenges for newly graduated nurses in the transition process. A study by Nour and Williams [34] revealed that owing to staffing shortages, NQRN/Ms in some settings were not paired with other nursing staff and were left to work alone. Similarly, Ho et al. [30] reported that many NQRN/Ms were under significant work pressure owing to staff shortages, absenteeism and the acute nature of patients’ illnesses.

Ancillary staff in healthcare settings provide supplemental or auxiliary services to patients in order to support their diagnosis and treatment [35]. This category includes porters, mortuary attendants, nursing assistants, cleaners, kitchen staff and laundry workers. The NQRN/Ms in this study indicated that they sometimes had to perform non-nursing duties owing to a lack of ancillary staff. Such duties included cleaning the floor in the labour ward after conducting a delivery or escorting patients to procedures such as X-rays. No reference was made by the NQRN/Ms to working in the kitchen and laundry rooms.

The findings further revealed that the NQRN/Ms are challenged by a lack of leadership skills, owing to their lack of experience. These findings are comparable to those of Willman, Bjuresäter and Nilsson [36], who reported that NQRN/Ms struggle with management and organisation, including managing their time, managing duty rosters and prioritising patients in complex situations. These limitations lead to them feeling stressed and less satisfied at work.

Inadequate supervision was also revealed to be a concern for the NQRN/Ms. Although becoming independent is important if they are to fully transition into their new roles [37], NQRN/Ms need some supervision during the transition period. The NQRN/Ms in the current study often felt unprepared and lacking in the necessary knowledge and skills that are required if they are to function well as a nurse. Graduate nurses thus believe that support from their colleagues and/or managers would be of great help to them [32]. The lack of supervision resulted in the NQRN/Ms in this study feeling unsafe and worried about making mistakes or causing problems for their patients, the hospital and themselves. This lack of support in the form of supervision may be because some more experienced nurses lack empathy and are not interested in supporting their less experienced colleagues [38]. Ho et al. [30] also reported a lack of supervision of NQRN/Ms, with nurses being in a ward on their first day with no orientation and no one to oversee their performance.

### Shortage of resources and infrastructure, and unreliable communication networks

Owing to a wide range of social, economic and political factors, most countries experience the disproportionate allocation of health resources, resulting in inequalities among population groups [39]. In particular, this disproportionate distribution leads to a lack of healthcare resources in rural areas. Studies conducted in Ghana, South Africa and Tanzania revealed that rural health districts experience a lack of resources, medical equipment and medicine, as well as poor maintenance of the resources and equipment that are available [40–42]. The current study similarly revealed a shortage of equipment and clinical supplies in the rural health district in which the NQRN/Ms were placed. Likewise, although their study was conducted with second-year nursing students, Nuuyoma and Ashipala [43] reported a limited supply of clinical stock and medication in a rural health centre in the southern part of Namibia. In the same way, limited resources were documented as hindrances to providing adequate support for rural graduate nurses in Western Australian [31].

While lengthy power outages are rare in high-income countries, rural communities in Africa are subjected to power outages on a daily basis [44]. Therefore, the finding on power outages as one of the experiences of the NQRN/Ms in the current study did not come as a surprise. Power outages negatively affect the provision of healthcare services, including hygiene and feeding apparatus. In some settings, healthcare facilities need heating or air conditioning equipment, life-support systems, monitoring and diagnostic apparatus, blood banks and transfusion services, morgue operations, and pharmaceutical and other supply storage that depend on electricity to function [45]. Power outages in health facilities can also lead to patient incivility, which has a significant negative effect on nurses’ stress levels [46].

Poor road networks were revealed in the current study as one of the challenges for NQRN/Ms in the rural health district. One of the signs of improperly maintained roads are potholes, which are often observed in rural areas owing to a lack of maintenance [40].

### Social life

The NQRN/Ms described themselves as being lonely and feeling isolated from their friends and family members. They also noted that there was a lack of opportunities for personal growth. Similarly, Ho et al. [30] found that NQRN/Ms have to make sacrifices in their home lives in order to stay in their jobs when they are far from their homes, families and friends. There is also a financial burden related to the cost of travelling to visit family and friends, as well as travelling to the capital or major towns where large shopping malls and central offices are located [40].

While some NQRN/Ms see their transition period as a time for growth [38], the current study revealed few opportunities for personal growth in the rural health district. This was owing to many factors, including being far from the capital and large towns, poor internet connectivity, no opportunities for continuing professional development activities, and limited access to further study opportunities as there are no institutions of higher education in the area. The feeling of professional isolation was described by Sandler et al. [47] as being a characteristic of rural and remote nursing. The opposite finding was reported by Smythe et al. [38], however, who indicated that NQRN/Ms were engaged in learning opportunities such as seminars, conferences and formal educational programmes.

Although participants in the current study did not mention any experiences related to the COVID-19 pandemic, recent literature has indicated that some nurses in rural health districts strengthened communication and collaboration within communities by becoming community educators, supporting their colleagues and becoming more resilient during the pandemic [48]. In other settings, however, rural nurses experienced role frustration owing to chaos in the care environment and feeling overwhelmed and abandoned by their communities, families and leaders during the pandemic [49].

### Strengths and limitations of the study

The strength of this study lies in the fact that data were collected from the NQRN/Ms themselves and not from secondary sources or non-NQRN/Ms. In addition, the study included participants who had completed their nursing training in different areas of the country; this created a heterogenous sample and therefore, the researchers were able to capture diverse experiences. The limitation of this study is that the participants did not include NQRN/Ms who were placed at outreach points and in remote clinics in the rural health district, as they were not accessible during data collection. It is possible that they would have highlighted other issues pertaining to placement in a rural health district that are not experienced at the district hospital and surrounding clinics. The study followed a qualitative, descriptive and explorative design, which was contextual in nature, therefore the generalisation of the findings to other contexts is limited. Moreover, the chosen data collection method of face-to-face interviews had the potential to limit responses from participants who were not comfortable conversing via a face-to-face mode rather than in a written format.

## Conclusion

It is concluded that NQRN/Ms in rural health districts have mixed experiences related to community members, colleagues, management, resources and their social lives. Generally, these findings may be used to improve Namibia’s undergraduate nursing curriculum, and to put in place graduate job preparation workshops and support networks at workplaces. In the rural context, the findings may assist nurse managers to plan and implement orientation sessions for NQRN/Ms, while in the international context the findings add to the body of knowledge on the experiences of NQRN/Ms in general rural health districts in developing countries. This may assist in understanding how limited resources, poor infrastructure and unreliable communication networks have impacted on NQRN/Ms on both the professional and the personal level.

It is recommended that rural health districts establish social networks and communities of practice for professionals for the purpose of sharing ideas, learning, general networking and participating in leisure activities and sports. Regular monitoring of NQRN/Ms should be undertaken by social workers, nurse managers or immediate supervisors, with referrals made for psychosocial support for those who show signs of loneliness, isolation and/or stress. NQRN/Ms should also be supported by means of the allocation of mentors or preceptors, as well as twinning with other NQRN/Ms. Moreover, health district management teams should engage with telecommunication network providers and village councils to improve roads and networks in rural areas.

## Electronic supplementary material

Below is the link to the electronic supplementary material.

Supplementary Material 1

Supplementary Material 2

## Data Availability

The data analysed during the study will be made available by the corresponding author upon reasonable request.

## List of abbreviations

NQRN/Ms: Newly Qualified Registered Nurses/Midwives
