# Supplementary material for: Newly qualified registered nurses’ and midwives’ experiences from rural health district placement in Namibia
Source: BMC Nurs. 2023 Apr 7;22:108. doi: 10.1186/s12912-023-01272-2 (PMC10080169; doi:10.1186/s12912-023-01272-2)
Supplement: Supplementary file 1 — Supplementary Material 1 [file 12912_2023_1272_MOESM1_ESM.pdf]

Supplementary material 1: Detailed data analysis record from quotations to themes

Participants' direct quotations	Codes	Sub-themes	Themes
"I was trained in another region and was never exposed to this people, so when I came to this area it was people from a different tribe that I have to deal with. I don't know their cultural background, I don't know what they like and what they dislike, so it was a big shock for me." [Participant 2]<br>"Sometimes people come to the hospital and want to perform some rituals, for me I don't know what it means, but for nurses who worked for longer periods in this district, they understand this people so well. I really don't know but I guess is just the beginning?" [Participant 6]	✓ People from different tribes<br>✓ Don't know cultural background<br>✓ Strange cultural rituals	✓ Cultural shock	1. Encounters with rural community members
"Yeah the challenge here, these people, I just want to be honest, you'll find someone……., the person is young, you can see this person can communicate in English but when you're talking to her she's responding in her language. Like I ask her in English, how can I help you	Patients respond in their languages<br>Patients do not want to speak English	✓ Language barrier	

today? She responded I know you're here for work, but you should talk our language. Imagine this is coming from a person who can speak English, but she doesn't not want to." [Participant 3]<br>"Sometimes a patient will come in the consulting room and throw the health card at you and then will just sit without saying anything. And then you end up asking in English and then that patient will say "no, you cannot speak to me in English, you should speak in my language." [Participant 4]<br>"It is difficult, there are old people who cannot speak English, since I do not speak local language, I sometimes have to seek assistance from colleagues to help with translation." [Participant 8]			
"Uh uh, the negative experiences would come from the community, not my colleagues rather. Community members make me feel like I don't belong here. because uh most of us that are placed here come from other districts, we	Don't belong in community<br>NQRN/Ms not one of their people<br>Outsiders	✓ Labelled as outsiders	

are not originally from here. So even before you open your mouth, or start to help the patient, they already have a negative mindset against you and so that's a very big disadvantage as a new person that comes here." [Participant 5]<br>"The moment they find out that you're not their tribe, and they will start insulting you and all that. They start discriminating against you, don't want to be treated by you because you are not one of their people." [Participant 4]			
"When I first came here, I was shy but then people were very friendly, they were very caring, and they warmly welcomed me. So, I really enjoying it. So, they are very helpful, they will be teaching you like this is done like this and that, and if you have any question, they are always available to answer your." [Participant 2]<br>"I was well received, my colleagues gave me detailed geographical orientation around the facility and also	Very caring colleagues<br>Warmly welcomed<br>Very helpful<br>Well received<br>Detailed geographical orientation<br>Good teamwork<br>Approachable and friendly staff	✓ Adequate support and teamwork	2. Encounters with colleagues

took me through some work procedures, I felt welcomed, I felt very welcomed. [Participant 7]<br>"I like how people work here, even if we're just two nursing staff on duty we make sure what needs to be done gets done." [Participant 5]<br>"There is good teamwork here, everyone is approachable and so friendly, even if you go to other departments in the hospital, people are friendly, and work well together." [Participant 4]			
"My colleagues were very surprised that I cannot perform some procedures, but I just completed my training, so I was not exposed to work industry yet apart from my clinical practice as a student." [Participant 1]<br>"Enrolled nurses had very high expectations from me which made me a bit uncomfortable, I must say." [Participant 8]	High expectations<br>Colleagues surprised as cannot perform procedures	✓ Unrealistic expectations	
"I worked alone at casualty department, and it was overloaded with patients, I think I received about 20 clients every night. When I go	Overloaded<br>Work alone<br>Perform non-nursing duties<br>Do porter's work<br>Do cleaner's work	✓ Insufficient nursing staffing and lack of ancillary staff	3. Staffing, management, and supervision

back home, I am very tired, even if I sleep, I still wake up very tired.” [Participant 1]<br>“I felt so disappointed, like how to do you let a nurse work alone in a whole facility which you know that all nearby villages will be coming to seek services there and I was alone doing everything, immunizations, initiations, family planning, treating, screening, you know all these things. It was really, I had a very terrible experience, I went to report, and they told me there’s nothing they can do about it, nurses are not enough.” [Participant 7]<br>“Basically, here I am working as a porter. Had to transport patients like from maternity department to outpatient department. Sometimes there’s a body that you have to transport to the mortuary in absence of the mortuary assistant and so on and we also do cleaning, as in some shifts there are no cleaners allocated, so it’s just too much.” [Participant 8]	Do mortuary attendant’s work		

"…… Although other categories like enrolled nurses are lower ranked than registered nurses, they worked more years than me and have accumulated a lot of experience. It is a problem managing them because I am still getting used to do off-duties schedule, delegation, and many others." [Participant 7]	Cannot manage enrolled nurses<br>Not used to do leadership tasks	✓ NQRN/Ms challenged with leadership skills	
"… I am a newly graduate nurse I don't know a lot of things and I work unsupervised at time and that's risky for me, to the patients and to the hospital itself, because if anything happens, it's going to be a problem." [Participant 2]<br>"When I started working here, were in the ward, all new………, we were two registered nurses but all working our probation period and no one to supervise." [Participant 3]	No one to supervise<br>Nurses on probation period scheduled alone together	✓ Inadequate supervision	
"We experience shortage of items like blood pressure monitoring machines, weighing scales, stethoscope, and others. Secondly resources such as medicine, there's	Medicines not in stock<br>Shortage of instruments for taking vital signs<br>No gloves<br>Ambulances not equipped	✓ Shortage of equipment and clinical supplies	4. Shortage of resources, infrastructures, and unreliable communication networks

literally every second day there's something that is not in stock, it's a real struggle." [Participant 7]<br>"Yeah, also you find yourself in the hospital, there's no gloves even for three days, four days and you're working with patient while you're in contact with bloods which also putting ourselves at risk." [Participant 3]<br>"Even ambulances here are not well equipped, there is no necessary ambulance equipment needed to transport patients from one point to another." [Participant 6]			
"Our electricity goes off a lot and is not good at all. When there is no electricity, the telephone network also doesn't work that means you cannot reach to the doctor on call if there is an emergency at the hospital. You also can't reach to the driver who is on call if you want assistance to get for you the doctor on call to come attend to emergency case" [Participant 3]<br>"Power failure for example and you have a patient that you	Electricity goes off<br>Power failure	✓ Power outages	

need to take for emergency caesarian section and there's no power or there's no way for you to communicate with the theater team, stuff like that." [Participant 5]<br>"if there is no electricity, everything is affected, maybe you want to read something on internet or consult online resources and do some other stuff is not possible. The moment it just rain here,…. even just a few drops, electricity goes off, internet goes off" [Participant 1]			
"The roads are not well constructed and perhaps not serviced from time to time? They get worse during rainy seasons when some of the areas are not accessible. Um yeah." [Participant 6]<br>"The roads are also so bad which makes the work difficult, you cannot travel freely especially with small vehicles." [Participant 8]	Roads not well constructed<br>Roads not conductive for small vehicles<br>Roads not safe<br>Roads not serviced<br>Bad roads	✓ Unsafe and bad roads	
"This is a rural area, there's no social life, you're stressed at work and there's nowhere to go for leisure activities just to	No social life<br>No leisure activities<br>No friends<br>Far from family	✓ Loneliness feeling and isolation from friends and family members	5. Experience on social life

relieve the stress and yeah.” [Participant 7]<br>“You cannot go visit your family members, you’re staying very far, you don’t have friends, so psychologically also you’re affected, if you have problems you cannot have anyone to talk to or maybe yeah, or maybe some therapy like maybe you need to go a movie theater just to relax your mind, it’s not available.” [Participant 6]<br>“Sometimes you feel like you are okay and sometimes on the other you feel like it’s far away from your family.” [Participant 1]<br>‘But on top of that, there are some of the challenges that I’ve met, as time goes on, I started feeling like, I’m so far I’m separated from my family since they are too far, and then I work from Monday to Friday, you won’t get enough time to go and visit your family members, it’s just Saturday and then Sunday you are coming back, it’s like you did not go there.[Participant 4]	Cannot visit family due to distance		

"…..and it's very difficult to travel, it requires transport money." [Participant 1]<br>"I feel I have extra financial burdens because if I have to travel, I have to spend a lot on transport." [Participant 8]	Requires transport money<br>Have extra financial burden<br>Spend lot of money on travelling	✓ Financial burden	
"If one wants to study further, it is not going to be easy at all because sometimes there no network so access to information available on internet." [Participant 1]<br>"News here reaches us even after three days, sometimes we don't even view other people's WhatsApp status because network is stripping." [Participant 3]	No opportunity to study further<br>No access to important information	✓ No opportunities for personal growth	
