# Supplementary material for: Newly qualified registered nurses’ and midwives’ experiences from rural health district placement in Namibia
Source: BMC Nurs. 2023 Apr 7;22:108. doi: 10.1186/s12912-023-01272-2 (PMC10080169; doi:10.1186/s12912-023-01272-2)
Supplement: Supplementary file 2 — Supplementary Material 2 [file 12912_2023_1272_MOESM2_ESM.pdf]

Supplementary Material 2: Interview protocol

Introduction

Researcher and participant read through the participant information sheet again (this is a refresher, participants are already introduced to the study at recruitment stage and consent to participate was obtained)

Obtain consent from participant for audio recording the interview

Obtain demographic information as follows;

Age

Sex

Marital status

Training institution where participant graduated from

Prior experience in health care settings

Confirm years/months of experience as a registered nurse/midwife (this is just to confirm for recording of demographic data, was already asked during recruitment stage to ensure inclusion criteria are followed)

Central question

What is your experience of placement in a rural health district as a newly qualified registered nurse/midwife?"

Probing questions*

*Probing depends on participant's response to the central question, probing used were *"tell me more about that, what do you mean by that? How did that made you feel?* However, the following two probing questions were asked to all participants

What was new or surprising for you? Why?

What was difficult or challenging? Why?

Closing question

We have come to an end of the interview, is there anything else you would like to add?
